# Long-term outcomes of second-line antiretroviral treatment in an adult and adolescent cohort in Myanmar

**DOI:** 10.1080/16549716.2017.1290916

**Published:** 2017-06-08

**Authors:** Nang Thu Thu Kyaw, Ajay M. V. Kumar, Myo Minn Oo, Htun Nyunt Oo, Khine Wut Yee Kyaw, Soe Thiha, Thet Ko Aung, Than Win, Yin Yin Mon, Anthony D. Harries

**Affiliations:** ^a^ The Union Office in Myanmar, International Union Against Tuberculosis and Lung Disease, Mandalay, Myanmar; ^b^ The Union South-East Asia Regional Office, International Union Against Tuberculosis and Lung Disease, New Delhi, India; ^c^ Research Department, International Union Against Tuberculosis and Lung Disease, Paris, France; ^d^ Department of Public Health, National HIV/AIDS Program, Nay Pyi Taw, Myanmar; ^e^ Department of Infectious and Tropical Diseases, London School of Hygiene and Tropical Medicine, London, UK

**Keywords:** HIV, virological failure, died, lost to follow-up, operational research

## Abstract

**Background**: Myanmar has a high burden of Human Immunodeficiency Virus (HIV) and second-line antiretroviral treatment (ART) has been available since 2008 in the public health sector. However, there have been no published data about the outcomes of such patients until now.

**Objective**: To assess the treatment and programmatic outcomes and factors associated with unfavorable outcomes (treatment failure, death and loss to follow-up from care) among people living with HIV (aged ≥ 10 years) receiving protease inhibitor-based second-line ART under the Integrated HIV Care Program in Myanmar between October 2008 and June 2015.

**Design**: Retrospective cohort study using routinely collected program data.

**Results**: Of 824 adults and adolescents on second-line ART, 52 patients received viral load testing and 19 patients were diagnosed with virological failure. However, their treatment was not modified. At the end of a total follow-up duration of 7 years, 88 (11%) patients died, 35 (4%) were lost to follow-up, 21 (2%) were transferred out to other health facilities and 680 (83%) were still under care. The incidence rate of unfavorable outcomes was 7.9 patients per 100 person years follow-up. Patients with a history of injecting drug use, with a history of lost to follow-up, with a higher baseline viral load and who had received didanosine and abacavir had a higher risk of unfavorable outcomes. Patients with higher baseline C4 counts, those having taken first-line ART at a private clinic, receiving ART at decentralized sites and taking zidovudine and lamivudine had a lower risk of unfavorable outcomes.

**Conclusions**: Long-term outcomes of patients on second-line ART were relatively good in this cohort. Virological failure was relatively low, possibly because of lack of viral load testing. No patient who failed on second-line ART was switched to third-line treatment. The National HIV/AIDS Program should consider making routine viral load monitoring and third-line ART drugs available after a careful cost–benefit analysis.

## Background

Globally, the number of HIV-positive patients accessing antiretroviral therapy (ART) has doubled since 2010 and 17 million people were on ART at the end of 2015 [1]. Mortality has reduced and the survival among HIV-positive patients has increased. Along with this benefit to people living with HIV. (PLHIV), the need to switch to second-line ART is also increasing because there are more patients who are spending longer periods of time on ART and failing on first-line ART. With more treatment experience on second-line ART, the numbers of patients failing this treatment and requiring third-line ART are increasing.

Different rates of second-line ART treatment failure have been reported. Studies from Asia reported that after two years on second-line ART, failure rates ranged between 8% and 41% [2–4] and studies from Africa reported that the rate was between 13% and 40% [3,5]. These studies have also described the different factors associated with second-line ART failure such as duration on first-line ART, late detection of first-line ART failure, current and prior ART regimens, age, body mass index and patient adherence on ART. Mortality rate was reported to be 13% at 5-year follow-up of a second-line ART cohort in Vietnam and 20% at 5-year follow-up in India [2,4]. High mortality in patients who are on second-line ART regimens and the challenges of managing this cohort of patients in terms of adherence to ART drugs, inadequate access to viral load monitoring and HIV genotype testing, and cost of access to third-line ART are of concern for programs treating PLHIV in resource-limited countries [6–8].

Myanmar is one of the high-HIV-burden countries in the South East Asia region with limited availability of HIV viral load testing and HIV drug resistance testing for monitoring patients who are on first-line as well as on second-line ART [9]. Currently, more than 100,000 patients (54% of all PLHIV) are receiving ART in Myanmar [9]. Although second-line ART has been available in the country since 2008, there are no published data on the number of patients on second-line ART, the characteristics of the cohort and their outcomes. With the limited availability of second-line ART. drug options and non-availability of third-line ART in the public health sector in Myanmar, it is important to understand the cohort of patients on second-line ART, their outcomes and factors associated with unfavorable outcomes on second-line treatment.

The Integrated HIV Care (IHC) Program has been implementing activities within the public sector via The Union Office in Myanmar, in collaboration with the National AIDS Program (NAP) and National TB Control Program (NTP) since 2005. The program has been providing treatment and chronic care for HIV-infected individuals in different parts of Myanmar, including second-line ART which has been available since 2008. This study reports on the treatment and programmatic outcomes of patients on second-line ART and the factors associated with unfavorable outcomes (treatment failure, death and loss to follow-up from care) among PLHIV who were on second-line ART after first-line ART failure in the IHC Program in Myanmar between October 2008 and June 2015.

## Methods

### Study design

This was a retrospective study of routinely collected program data.

### Study setting

Myanmar is one of the high-HIV-burden countries with limited resources. The country is located in Southeast Asia and administratively divided into 15 regions. The Union’s IHC Program works with the NAP in the treatment and care of PLHIV at 33 sites in Mandalay, Sagaing, Magway, Shan and Yangon regions. It has been providing HIV care to about 30,000 patients since 2005, and about 23,000 patients are currently on ART under this program. The HIV management and ART provision at IHC sites follow the NAP guidelines [10] and all services including ART drugs and laboratory investigations are provided free of charge. A protease inhibitor-based second-line ART has been available in the program since 2008. Currently, the national program recommends second-line ART comprised of zidovudine (ZDV) or tenofovir (TDF), lamivudine (3TC), and ritonavir-boosted lopinavir (LPV/r) for adults and adolescents. Before 2013, stavudine (D4T) and didanosine (ddI) were used instead of ZDV, TDF or abacavir (ABC).

Patients on the second-line ART regimens are monitored clinically and immunologically. The program follows the N.A.P guidelines for diagnosing clinical, immunological and virological failure which are based on World Health Organization (WHO) guidelines [10,11]. Clinical failure is diagnosed by the clinician if a patient develops a new or recurrent WHO clinical stage III or IV condition after having been on ART for 6 months or longer. Immunological failure is diagnosed if the CD4 cell count falls to the baseline or below, or stays persistently below 100 cells/µl after 6 months on ART. Routine HIV viral load monitoring has not been available yet in this setting. The program follows a system of targeted viral load testing by which HIV viral load testing is done when the patient is suspected to have clinical and/or immunological failure. Virological failure is diagnosed if a patient’s viral load is greater than 5000 copies/ml (before March 2015) or greater than 1000 copies/ml (after March 2015).

Viral load testing is carried out using an automated c1000 RealTime PCR system (Bio-Rad Sciences, Hercules, California, USA and HIV Generic Charge Virale, Biocentric, Bandol, France) at the Public Health Laboratory or using Rotor-Gene Real-Time Analysis (PG. Biotech HIV detection kit) at an outside laboratory if the in-house viral load machine is not available. The CD4-count is measured by cyflow cytometry (Pertec-sysmex, Wakinohama-kaigandari, Japan). HIV genotyping and drug resistance testing are not accessible for routine practice and not available in the country. The third-line ARV drugs such as ritonavir-boosted darunavir (DRV) and atazanavir (ATV), etravirine (ETR) and raltegravir (RAL), which are recommended in the NAP 2014 guidelines [10], are not available in the program. However, there are a few patients who are on third-line ART as a result of their own out-of-pocket expenditure for those drugs.

### Study population

We included all adult (age > 19 years) and adolescent (between 10 and 19 years of age) PLHIV who initiated protease inhibitor-based second-line treatment due to documented clinical, immunological and/or virological failure on first-line ART under the IHC Program between 1 October 2008 and 31 June 2015. We excluded patients who started second-line ART before being registered to the IHC Program or who were on protease inhibitors but did not have documentation of first-line ART failure.

### Variables

The data variables for the study included age, gender, occupation, literacy, current sites of provision of second-line ART, ART regimens, date of starting first-line and second-line ART, clinical staging, CD4 counts and viral load result at the time of switching to second-line ART, viral load (if available) on second-line ART, date of CD4 and viral load testing, lost to follow-up history, co-morbidities (Hepatitis B and C), date of death, date of diagnosis of virological failure, date of loss to follow-up, numbers of times the first-line ART regimens were changed, duration on treatment (first-line and second-line) and delayed switching to second-line ART.

Duration on first-line ART was defined as the time between the dates of starting first-line ART and starting second-line ART. Duration on second-line ART was defined as the time between the dates of start of second-line ART and outcomes. Patients were considered to have had a ‘delayed switching to second-line ART’ if the duration between date of virological failure on first-line ART and date of starting second-line ART was more than four months. A cutoff of four months was used because as per national protocol, a first viral load result indicating failure criteria was followed by intensive adherence counseling for three months and a repeat viral load test was done before making the switch.

The treatment outcome variables include virologically failed and not failed. The programmatic outcome variables include regular follow-up (attending clinic appointment regularly), death, lost to follow-up, and transfer out to other facilities. As mentioned earlier, we defined virological failure as a viral load result greater than 5000 copies/ml before March 2015 and greater than 1000 copies/ml after March 2015. We used the most recent date of a viral load result that met the criteria for virological failure as the date of diagnosis of virological failure. Death was recorded if the patient’s family or the outreach workers reported to the clinic that the patient had died. Cause of death was not systematically recorded in this setting. Lost to follow-up was defined as the patient not attending the clinic within three months after the scheduled appointment date.

We also categorized overall unfavorable outcome which included patients who were categorized as virological failure, dead or lost to follow-up. Patients who were retained in care and transferred out to other facilities without having virological failure were categorized as having favorable outcomes. For patients who died or were lost to follow-up or transferred out, the date of the last appointment before the event was considered as the censor date. If a patient was lost to follow-up from the program for more than three months and then came back to the program, we considered that he/she was ‘lost to follow-up at-least once’. If a patient was lost to follow-up from care more than once, the date of the most recent lost to follow-up date was used as the censor date. Patients who were on regular follow-up care without any other event were categorized as having favorable outcomes and 31 December 2015 was considered as the censor date.

### Sources and collection of data

The data were all stored at the electronic patient database which is regularly maintained and updated. Data required for this study were extracted to a Microsoft *Excel* file after removing patients’ identifiers between February 2016 and April 2016.

### Statistical analyses

The cumulative incidence (95% confidence interval [CI]) of virological failure, death and loss to follow-up was calculated using Kaplan–Meier methods. The factors associated with unfavorable outcomes (virological failure, dead and lost to follow-up) were analyzed using the Cox proportional hazard model. Variables with a *p*-value < 0.2 in univariate analysis were included in the multivariate model and adjusted Hazard Ratios (HR) were calculated. Records with missing covariates were excluded in the multivariate model. Statistical analyses were performed using Stata software [12].

## Results

Between October 2008 and June 2015, 1538 adult and adolescent patients were receiving ritonavir-boosted lopinavir (LPV/r)-based second-line ART, out of which 281 patients were already on LPV/r before getting registered to IHC, 13 patients switched to LPV/r due to toxicity and for 420 patients, no documentation on reason for switching was available. Thus, only 824 patients who were switched from first-line ART due to treatment failure were included in this cohort analysis.

### Characteristics of the cohort

A total of 824 adult and adolescent patients with mean age of 37 years (Standard deviation [S.D] 9 years) switched to second-line ART due to failure on first-line ART. Baseline and follow-up characteristics of this second-line cohort are shown in Table 1 . About 65% were male and only 5% were from the adolescent age group. Most were married, literate and employed. Sixty-four percent of patients had a history of taking ART at the private provider. Median and interquartile range (IQR) duration on first-line ART at IHC before switching to second-line ART was 27 (IQR 17–40) months. Date of first-line ART failure was available for 761 patients with median duration between failure on first-line ART and initiation of second-line treatment being 2 (IQR 1–3) months. Delayed switching occurred in 12% of them. Only 20% of the patients had a history of being lost to follow-up under IHC care.

Baseline CD4 counts were available for 823 (99%) patients and baseline HIV viral load tests were available for 560 (68%) patients. At the start of second-line treatment, patients had a median CD4 count of 115 (IQR 62–213) cells/μl and HIV-1 RNA of 4.8 (IQR 4.2–5.3) log10 copies/ml. Most of the patients (77%) received tenofovir (TDF) as part of their second-line ART regimen. All second-line regimens were based on LPV/r. Two patients were prescribed ritonavir-boosted raltegravir (RAL/r) and etravirine (ETV) combined with LPV/r as part of their second-line regimen. Median follow-up duration on second-line ART was 24 (IQR 12–34) months. The maximum duration of second-line treatment of the patients who were still under care without virological failure was 7 years.

### Virological failure on second-line ART

**Figure 1.**
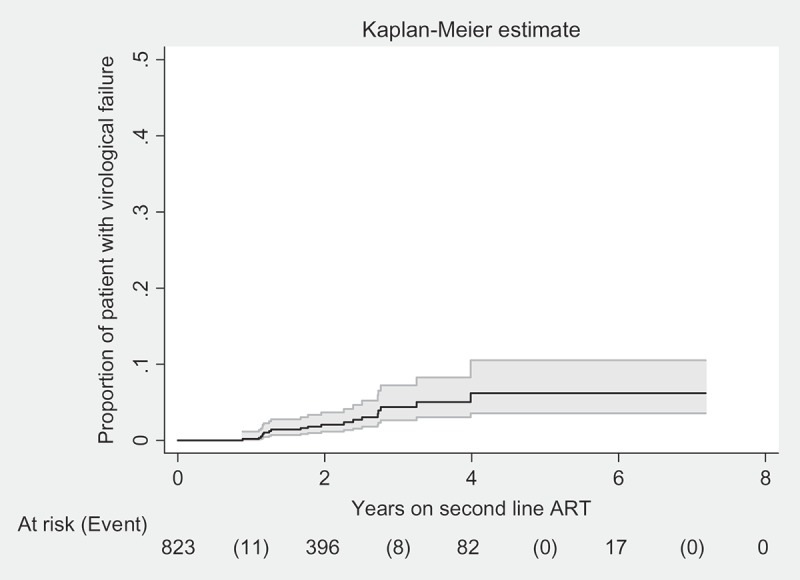
Number of patients and cumulative probability of virological failure in second-line ART cohort at IHC, Myanmar between 2008 and 2015.

Of 824 patients, 52 (6%) patients received viral load testing after initiation of second-line treatment during the study period and 19 (37% of those tested) were diagnosed with virological failure according to WHO criteria. None of these 19 patients switched to third-line ART during the study period. The incidence rate of virological failure after starting second-line ART was 1 patient per 100 person years follow-up (PYFU) (95% CI: 0.7–1.7). The cumulative probability of virological failure at year 7 was 6% (95% CI: 3–9%) (Figure 1).

### Program outcomes

**Figure 2.**
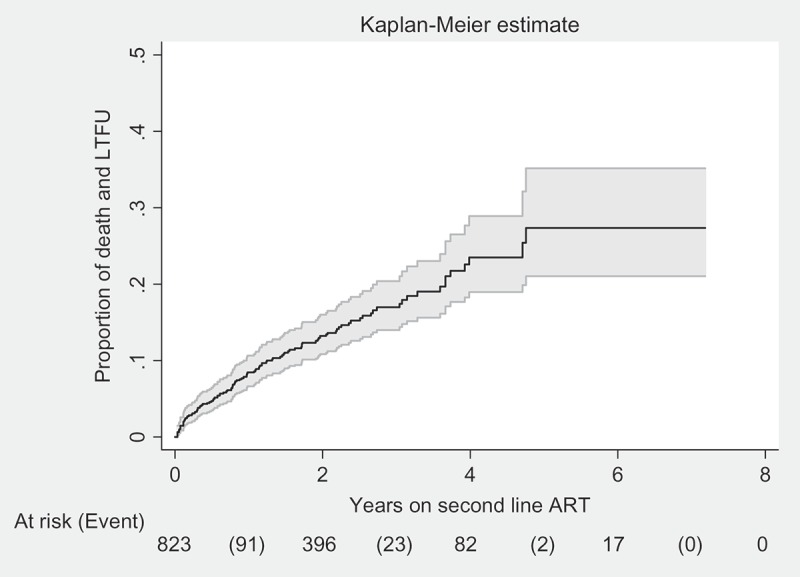
Number of patients and cumulative probability of death and lost to follow-up combined in second-line ART cohort at IHC, Myanmar between 2008 and 2015.

Among 824 patients on second-line ART, 88 patients (11%) died, 35 (4%) patients were lost to follow-up, 21 (2%) were transferred out to other health facilities and 680 (83%) were retained under care at the end of the study period. Of 88 deaths, 48 (55%) patients died within 1 year of starting second-line ART. Of 35 patients lost to follow-up, 17 (48%) were also lost to follow-up within 1 year. Of 19 patients who experienced virological failure, 12 patients were still under care, 1 patient was transferred out, 5 patients died and 1 patient was lost to follow-up at the end of the study period. The incidence rate of death and lost to follow-up combined was 7 patients per 100 PYFU (95% CI: 6–8) and the cumulative probability of death and lost to follow-up at year 7 was 26% (95% CI: 20–33) (Figure 2).

**Figure 3.**
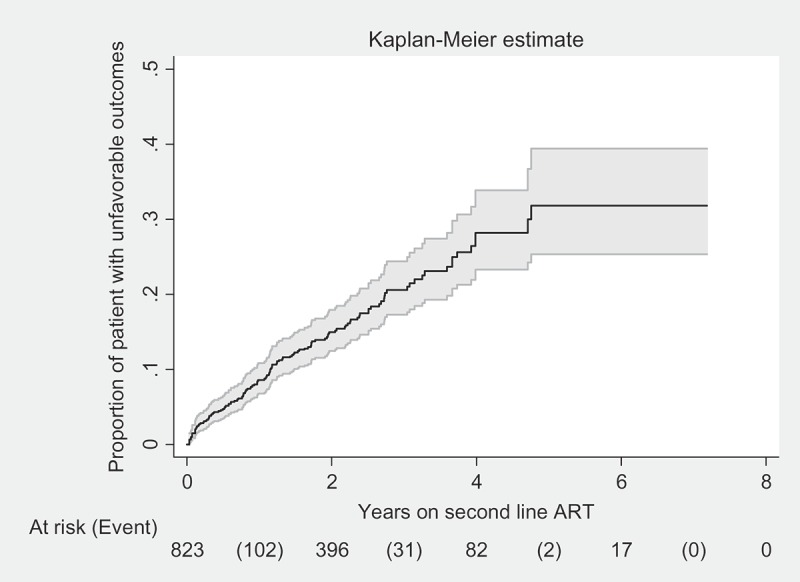
Number of patients and cumulative probability of unfavorable outcomes (failure, death and lost to follow-up combined) in second-line ART cohort at IHC, Myanmar between 2008 and 2015.

Overall, 136 (17%) patients’ outcomes were unfavorable (failure, death, lost to follow-up) at the end of the study period. The probability of an unfavorable outcome at year 1 was 8% (95% C.I: 7–11), at year 2 it was 15% (95% CI: 13–18) and at year 7 it was 30% (95% CI: 25–37%) (Figure 3); the incidence rate of unfavorable outcomes across the study period was 7.9 per 100 PYFU (95% CI: 6.7–9.3). This rate was different among sub-groups (Table 2).

**Table 1.** Socio-demographic and clinical characteristics of patients who started second-line ART at the IHC Program, Myanmar between 2008 and 2015, stratified by treatment outcomes.

Socio-demographic characteristics		Failure	Death/Lost to follow-up	Favorable outcome	Total
		n (%)	n (%)	n (%)	n (%)
All patients					824 (100)
Gender	Male	10 (53)	85 (73)	437 (64)	532 (65)
	Female	9 (47)	32 (27)	251 (36)	292 (35)
Age group	Adolescent (10–19 years)	3 (16)	4 (3)	35 (5)	42 (5)
	Adult (> 19 years)	16 (84)	113 (97)	653 (95)	782 (95)
Marital status	Single	7 (37)	35 (30)	181 (26)	223 (27)
	Married	8 (42)	56 (48)	372 (54)	436 (53)
	Widowed	4 (21)	16 (14)	103 (15)	123 (15)
	Divorced/separate	0 (0)	6 (5)	23 (3)	29 (4)
	Missing	0 (0)	4 (3)	9 (1)	13 (2)
Employment status	Employed	11 (58)	83 (71)	484 (70)	578 (70)
	Unemployed	8 (42)	33 (28)	198 (29)	239 (29)
	Missing	0 (0)	1 (1)	6 (1)	7 (1)
Literacy	Literate	13 (68)	111 (95)	615 (89)	739 (90)
	Illiterate	6 (32)	5 (4)	68 (10)	79 (10)
	Missing	0 (0)	1 (1)	5 (1)	6 (1)
HIV transmission risk	Heterosexual	13 (68)	92 (79)	573 (83)	678 (82)
	Men sex with men	0 (0)	4 (3)	16 (2)	20 (2)
	Sex work	0 (0)	0 (0)	1 (0)	1 (0)
	Injecting drug use	1 (5)	8 (7)	14 (2)	23 (3)
	Blood transfusion	2 (11)	6 (5)	24 (3)	32 (4)
	Mother to child	3 (16)	2 (2)	32 (5)	37 (4)
	Unknown	0 (0)	5 (15)	28 (85)	33 (4)
Caretaker	Yes	18 (95)	84 (72)	528 (77)	630 (76)
	No	1 (5)	33 (28)	160 (23)	194 (24)
Region of ART clinic	Mandalay	16 (84)	89 (76)	444 (65)	549 (67)
	Sagaing	0 (0)	6 (5)	64 (9)	70 (9)
	Magway	1 (5)	4 (3)	73 (11)	78 (9)
	Shan	0 (0)	15 (13)	55 (8)	70 (9)
	Yangon	2 (11)	3 (3)	52 (8)	57 (7)
On ART before enrolled to IHC	Private	19 (100)	78 (67)	432 (63)	529 (64)
	Public	0 (0)	29 (25)	198 (29)	227 (28)
	Unknown place	0 (0)	9 (8)	44 (6)	53 (6)
	Naïve	0 (0)	1 (1)	14 (2)	15 (2)
Baseline characteristics					
Duration on first-line ART	< 1 year	1 (5)	16 (14)	74 (11)	91 (11)
	1–2 years	6 (32)	45 (38)	211 (31)	262 (32)
	> 2 years	12 (63)	56 (48)	403 (59)	471 (57)
First-line ART modification (times)	No change	6 (32)	55 (47)	377 (55)	438 (53)
	Three times	13 (68)	60 (51)	287 (42)	360 (44)
	More than three times	0 (0)	2 (2)	24 (3)	26 (3)
Lost to follow-up (number of times)	No lost to follow-up	12 (63)	81 (69)	564 (82)	657 (80)
	1 time	4 (21)	29 (25)	101 (15)	134 (16)
	> 1 time	3 (16)	7 (6)	23 (3)	33 (4)
Delayed switching to second-line ART	Within 4 months	15 (79)	92 (79)	560 (81)	667 (81)
	Delayed	1 (5)	14 (12)	79 (12)	94 (11)
	Missing failure date	3 (16)	11 (9)	49 (7)	63 (8)
C.D.4 count (cells/µl)	More than 350	2 (11)	4 (3)	71 (10)	77 (9)
	Between 100 and 350	4 (21)	39 (33)	336 (49)	379 (46)
	Less than 100	13 (68)	74 (63)	280 (41)	367 (45)
	Missing	0 (0)	0 (0)	1 (0)	1 (0)
WHO staging	Stage I/II	4 (21)	28 (24)	175 (25)	207 (25)
	Stage III/IV	15 (79)	89 (76)	513 (75)	617 (75)
Viral load (copies/ml)	< 10,000	0 (0)	9 (8)	65 (9)	74 (9)
	> 10,000	12 (63)	67 (57)	407 (59)	486 (59)
	Missing	7 (37)	41 (35)	216 (31)	264 (32)
Hepatitis B	Negative	19 (100)	90 (77)	587 (85)	696 (84)
	Positive	0 (0)	18 (15)	68 (10)	86 (10)
	Missing	0 (0)	9 (8)	33 (5)	42 (5)
Hepatitis C	Negative	19 (100)	102 (87)	627 (91)	748 (91)
	Positive	0 (0)	7 (6)	28 (4)	35 (4)
	Missing	0 (0)	8 (7)	33 (5)	41 (5)
Follow-up characteristics					
Current ART site	ART Center	18 (95)	106 (91)	459 (67)	583 (71)
	Decentralized site	1 (5)	11 (9)	229 (33)	241 (29)
Duration on second-line ART	< 1 year	0 (0)	65 (56)	153 (22)	218 (26)
	1–2 years	3 (16)	27 (23)	172 (25)	202 (25)
	> 2 years	16 (84)	25 (21)	363 (53)	404 (49)
NRTI class	TDF-based	17 (89)	88 (75)	528 (77)	633 (77)
	d.d.I+ABC	1 (5)	12 (10)	20 (3)	33 (4)
	ABC+3T.C	1 (5)	6 (5)	51 (7)	58 (7)
	AZT+3T.C	0 (0)	8 (7)	84 (12)	92 (11)
	d4T+3T.C	0 (0)	2 (2)	3 (0)	5 (1)
	ETV	0 (0)	1 (1)	1 (0)	2 (0)
	Missing	0 (0)	0 (0)	1 (0)	1 (0)

*Notes:* ART = antiretroviral treatment; WHO = World Health Organization; IHC = Integrated HIV Care Program; NRTI = nucleoside reverse-transcriptase inhibitors; NNRTI = non-nucleoside reverse-transcriptase inhibitors; TDF = tenofovir; d.d.I = didanosine; ABC = abacavir; AZT = zidovudine; 3TC = lamivudine; d4T = stavudine; ETV = etravirine.

n (%) = number (percentage). Percentages are column percentages.

**Table 2.** Incidence rates of unfavorable outcomes among patients on second-line ART in the IHC Program, Myanmar between 2008 and 2015.

Baseline characteristics		Rate (95% CI) per 100 PYFU
	Total	7.9 (6.7–9.3)
Gender	Male	6.9 (5–9.3)
	Female	8.4 (6.9–10.3)
Age group	Adult	7.8 (6.5–9.3)
	Adolescent	10.4 (5–21.8)
Transmission risk	Heterosexual	7.2 (5.9–8.7)
	Men sex with men	8 (3–21.2)
	Injecting drug use	24.5 (12.8–47.2)
	Blood transfusion	12.3 (6.2–24.6)
	Mother to child	7.9 (3.3–19)
	Unknown	10 (4.1–23.9)
Caretaker	Yes	8.3 (6.9–10.1)
	No	6.8 (4.8–9.6)
ART before enrolled	Private	5.2 (3.6–7.6)
	Public	6.2 (3.2–11.9)
	Unknown	2.5 (0.3–17.6)
	Naïve	9.8 (8–12)
Duration on first-line ART	< 1 year	6.8 (4.1–11.1)
	1–2 years	9.5 (7.2–12.5)
	> 2 years	7.2 (5.7–9.2)
First-line ART modification (times)	No Rx changed	7 (5.4–9)
	1–3 times	9 (7.2–11.3)
	> 3 times	4.4 (1.1–17.4)
Lost to follow-up (number of times)	No lost to follow-up	6.7 (5.4–8.2)
	1 time	12.7 (9–17.9)
	> 1 times	13.7 (7.4–25.5)
Delayed switching to second-line ART	Switch within 4 months	7.4 (6.1–8.9)
	Delayed	10.8 (7.4–15.6)
C4 count (cells/µl)	More than 350	4.7 (2.1–10.5)
	Between 100 and 350	5.3 (3.9–7.2)
	Less than 100	11.1 (9–13.7)
WHO staging	Stage I/II	7.8 (5.5–11.1)
	Stage III/IV	7.9 (6.5–9.6)
Viral load (copies/ml)	< 10,000	5.6 (2.9–10.8)
	> 10,000	7.7 (6.2–9.6)
	Missing	8.9 (6.7–11.8)
Hepatitis B	No	7.6 (6.3–9.1)
	Yes	8.6 (5.4–13.8)
	Missing	12.2 (6.3–23.4)
Hepatitis C	No	7.7 (6.4–9.2)
	Yes	9.2 (4.4–19.4)
	Missing	10.9 (5.4–21.8)
Current ART site	ART sites	12 (10.1–14.4)
	D.C sites	1.7 (1–3.1)
Duaration on second-line ART	< 1 year	58.6 (45.9–74.9)
	1–2 years	9.8 (6.8–14)
	> 2 years	3.2 (2.3–4.3)
NRTI and NNRTI class	TDF-based	7.5 (6.2–9.1)
	d.d.I+ABC	8.6 (5–14.8)
	ABC+3TC	8.4 (4–17.6)
	AZT+3TC	11.1 (5.6–22.3)
	D4T+3TC	18.5 (4.6–74)
	ETV	59 (8.3–418.9)

*Notes:* PYFU = person years follow-up; ART = antiretroviral treatment; WHO = World Health Organization; IHC = Integrated HIV Care Program; NRTI = nucleoside reverse-transcriptase inhibitors; NNRTI = non-nucleoside reverse-transcriptase inhibitors; TDF = tenofovir; ddI = didanosine; ABC = abacavir; AZT = zidovudine; 3TC = lamivudine; d4T = stavudine; ETV = etravirine.

### Factors associated with unfavorable outcomes

Factors associated with unfavorable outcomes are shown in Table 3. In unadjusted analysis, patients with a history of injecting drug use, a history of being lost to follow-up, having a higher baseline viral load and who received ddI+ABC as the NRTI (nucleoside reverse-transcriptase inhibitors) drug class in the lopinavir-based regimen had a significantly higher risk of unfavorable outcomes. Patients with higher CD4 cell counts at baseline, having taken first-line ART at a private clinic, receiving ART at decentralized sites, having a longer duration on second-line ART and taking ZDV+3TC had a significantly lower risk of unfavorable outcomes. In the adjusted analysis, receiving ddI+ABC, duration on second-line ART and taking ART at decentralized sites still had statistically significant associations.

**Table 3.** Factors associated with unfavorable outcomes in patients on second-line ART in the IHC Program, Myanmar between 2008 and 2015.

Characteristics		HR (95% CI)	*p*-value	aR (95% CI)	*p*-value
Gender	Female	Ref			
	Male	1.2 (0.8–1.7)	0.36		
Age group (years)	Adult	Ref			
	Adolescent	1.3 (0.6–2.7)	0.55		
Transmission risk	Heterosexual	Ref		Ref	
	Men sex with men	1.2 (0.4–3.1)	0.78	1.2 (0.3–4.6)	0.75
	Injecting drug use	3.2 (1.6–6.4)	< 0.001	1.7 (0.7–3.9)	0.22
	Blood transfusion	1.7 (0.8–3.5)	0.14	1.1 (0.3–3.8)	0.82
	Mother to child	1 (0.4–2.6)	0.93	0.2 (0–1.4)	0.10
	Unknown	1.3 (0.5–3.3)	0.54	0.9 (0.3–2.7)	0.89
Caretaker	Yes	Ref			
	No	0.8 (0.6–1.2)	0.36		
ART before enrolled	Private	0.5 (0.4–0.8)	0.01	0.7 (0.4–1.2)	0.19
	Public	0.6 (0.3–1.3)	0.21	1.7 (0.6–4.6)	0.30
	Unknown place	0.3 (0–1.9)	0.19	1.5 (0.2–11.5)	0.69
	Naïve	Ref		Ref	
First-line ART modification (times)	No change	Ref		Ref	
	Three times	1.3 (0.9–1.9)	0.10	1.5 (1–2.4)	0.08
	More than three times	0.7 (0.2–2.7)	0.57	0.6 (0.1–4.2)	0.57
Lost to follow-up (number of times)	No lost to follow-up	Ref		Ref	
	1 time	1.9 (1.3–2.8)	< 0.001	1 (0.6–1.7)	0.92
	> 1 time	2.1 (1.1–4)	0.03	1.3 (0.5–3.2)	0.57
Delayed switching to second-line ART	Switch within 4 months	Ref		Ref	
	Delayed	1.4 (1–2.2)	0.08	0.5 (0.1–1.8)	0.29
WHO staging	Stage I/II	Ref			
	Stage III/IV	1 (0.7–1.5)	0.85		
C4 count (by + 10 cells/µl)		0.97 (0.95–0.99)	< 0.001	0.98 (0.96–1)	0.07
Viral load (by + log10 copies/ml)		1.6 (1.2–2.1)	< 0.001	1.2 (0.9–1.6)	0.31
Follow-up clinical characteristics					
Current ART site	ART Center	Ref		Ref	
	Decentralized site	0.1 (0.1–0.3)	< 0.001	0.3 (0.1–0.7)	< 0.01
Duration on second-line ART (every 1 year increased)		0.6 (0.5–0.6)	< 0.001	0.6 (0.5–0.6)	< 0.001
NRTI and N.NRTI class	TDF-based	Ref		Ref	
	d.d.I+ABC	1.5 (0.8–2.7)	0.21	4 (1.6–9.7)	< 0.01
	ABC+3TC	1.1 (0.5–2.3)	0.88	0.6 (0.2–1.8)	0.37
	AZT+3TC	1.3 (0.6–2.7)	0.49	0.3 (0.1–0.9)	0.03
	d4T+3TC	2.5 (0.6–10.2)	0.20	1.5 (0.3–6.9)	0.63
	ETV	7.4 (1–53.6)	0.05	9.1 (0.8–98.6)	0.07

Notes: HR = hazard ratio; aHR = adjusted hazard ratio; 95% CI = 95% confidence interval; ART = antiretroviral treatment; WHO = World Health Organization; IHC = Integrated HIV Care Program; NRTI = nucleoside reverse-transcriptase inhibitors; NNRTI = non-nucleoside reverse-transcriptase inhibitors; TDF = tenofovir; ddI = didanosine; ABC = abacavir; AZT = zidovudine; 3TC = lamivudine; d4T = stavudine; ETV = etravirine.

## Discussion

This study is the first to report on the outcomes of a second-line ART cohort of patients being managed in a public health setting in Myanmar. Treatment failure was suspected based on clinical and immunological criteria and only these patients received viral load testing. The WHO recommends systematic viral load testing for all patients (at sixth month after treatment and yearly thereafter to monitor virological response) [11]. However, there is no systematic viral load testing available in this setting and hence the number of patients who received viral load testing was lower than the numbers that should have been tested.

The failure rate and overall outcomes of this patient cohort are better than in other studies from similar resource-limited settings [2,5,7,8,13]. A multi-centric study with follow-up duration of three years showed that the probability of survival at one year was 0.86 and at two years it was 0.77 [8]. A study from Vietnam reported that 9.5% of patients on second-line ART had failure during 6-year follow-up [14]. There were substantial early mortality and loss to follow-up in this study but the outcomes are better in the later years among the survivors. This result is consistent with a study from India which is similar to our study setting where routine viral load monitoring was not available. This India study showed that the death rate was higher in the first year (13.7 patients per 100 PYFU in first year vs 3.9 patients per 100 PYFU between 1 and 5 years after second-line treatment) mainly due to the longer duration of patients failing on first-line ART as a result of delays in failure diagnosis using clinical and immunological criteria and having poor clinical condition at the start of second-line ART [2].

Adolescents only accounted for 5% of this second-line ART cohort although the evidence suggests that the adolescent age group is more likely to fail on first-line ART due to complex psychosocial, behavioral and clinical reasons [14–17]. More research is needed in Myanmar to explore whether the HIV-infected adolescents lack adequate access to care or if the care is not tailored to their age group.

Myanmar has a concentrated HIV epidemic. The HIV burden was very high in key populations with HIV prevalence rates ranging from 6% to 23% while HIV prevalence in the general population was 0.5% in 2015 [18]. In addition, key populations are more likely to experience treatment failure and unfavorable outcomes [19,20]. However, our result showed that only 5% of the second-line ART cohort belonged to key populations (commercial sex workers, men who have sex with men and injection drug users). This might be because a large proportion of key populations are not accessing HIV testing services in the first place, not starting on HIV treatment, not having access to second-line treatment or not disclosing their real HIV risk to their care provider [21,22]. All these issues need to be further explored in order to better understand and perhaps modify the services provided to these key populations.

One in five patients in this cohort experienced delayed switching to second-line treatment. This delay in treatment is not uncommon in many resource-limited countries [23,24]. Treatment providers are often reluctant to switch early due to limited diagnostic tools such as HIV viral load testing and drug resistance testing, the difficulties in ensuring that patients are adherent to medication and the availability and costs of the second-line drugs [25].

Although more than half of this cohort was on ART for more than 2 years, only 6% of patients were tested for HIV viral load after 6 months of second-line therapy. This can explain the lower rates of virological failure and may indicate the under-diagnosis of second-line treatment failure. In addition, the evidence suggests that most of the second-line treatment failure cases are not actually resistant to second-line therapy in the initial years of treatment and they have high viral loads due to problems with drug adherence [26].

Among the 19 patients who met the virological failure criteria, none of them were switched to a third-line regimen. As there is no information on their patterns of HIV drug resistance, it is difficult to know whether their therapy was not modified because it was deemed clinically unnecessary to switch or because the third-line ART regimen was not available in the program. This underscores the need for HIV drug resistance testing in Myanmar for those patients diagnosed with second-line ART failure and the need for third-line ART for those with confirmed resistance to second-line ART to prevent further transmission of resistant HIV.

Risk factors for unfavorable outcomes in our own study and in other studies have consistently included high viral load and low CD4 counts at baseline, having a history of being lost to follow-up and experiencing delayed switching to second-line ART after first-line failure [2,7,8,13,25]. Although there were small numbers of patients who were injection drug users in our study, they have a higher risk of developing unfavorable outcomes similar to what has been shown elsewhere [21].

In this cohort, about one third of patients receiving second-line ART were at a decentralized site and they had better outcomes than people in the ART Center. The probable reason is that these patients had good ART outcomes in general because only those who are clinically stable are referred to a decentralized site. This highlights the success of the decentralization strategy by the national program to bring the treatment including second-line treatment nearer to the patients’ residence for easy access.

We also noted that patients who had a history of receiving treatment at private clinics before coming to IHC had a better outcome. There is no study comparing outcomes between the public and private sectors. However, we assume that the patients who seek care from the private sector are more likely to be well-off economically and hence might have maintained better adherence to the treatment.

### Strengths

This study had a large sample size with a long duration of follow-up of patients on second-line ART in comparison with other studies. The study was based on routinely collected public health program data which can reflect the reality of second-line ART management in a resource-limited setting. We used standard virological failure and other programmatic outcomes definitions in accordance with WHO guidelines, enabling comparisons with the findings from other studies. We had robust and reliable data with dates of outcomes for each patient, thus enabling time-to-event analysis. We also reported this study according to the Strengthening the Reporting of Observational Studies in Epidemiology (STROBE) guidelines [27].

### Limitations

There is risk of selection bias as we excluded patients who were on a LPV/r-based regimen with no documentation of the reason for switching from first-line ART to a LPV/r-based regimen and who were already on a LPV/r-based regimen when they registered at IHC. In addition, there is no information on clinical and immunological failure diagnosed by the clinician in patients on second-line ART who did not receive viral load testing. Hence, there may be an underestimation of the treatment failure rate in this study. A few patients had had HIV drug resistance testing done with out-of-pocket expenditure but the results were not documented in this database. We do not have data on cause of death and cannot therefore determine if the death was HIV-related or not.

## Conclusions

Long-term outcomes of patients on second-line ART were relatively good in this cohort. Most of the deaths and loss to follow-up occurred in the first year of treatment. A low percentage was found to have virological failure because only a very small percentage of patients was tested for viral load. Thus, routine viral load monitoring is recommended for the cohort of second-line ART patients. Finally, the National HIV/AIDS Program should consider making available third-line ART drugs for such patients after a careful cost–benefit analysis.

## Paper context

While second-line antiretroviral treatment (ART) has been available since 2008 in Myanmar, there has not yet been an assessment of patient outcomes. The rate of unfavorable outcomes (failure, death, lost-to-follow-up) among 824 patients was relatively low at 7.9 per 100 person-years. However, only 52 received HIV viral load tests and 19 who failed treatment were not changed to third-line ART. The Myanmar National HIV/AIDS Program should consider making routine viral load monitoring and third-line ART drugs available.
